# Identifying nonprescription antibiotic users with screening questions in a primary care setting

**DOI:** 10.1017/ash.2023.238

**Published:** 2023-09-29

**Authors:** Eva Amenta, Marissa Valentine-King, Lindsey Laytner, Michael Paasche-Orlow, Richard Street, Kenneth Barning, Thomas Porter, Hammad Mahmood, Barbara Trautner, Larissa Grigoryan

## Abstract

**Background:** Antibiotic use without a prescription (nonprescription use) leads to antibiotic overuse, with negative consequences for patient and public health. We studied whether screening patients for prior nonprescription antibiotic use in the past 12 months predicted their intentions to use them in the future. **Methods:** A survey asking respondents about prior and intended nonprescription antibiotic use was performed between January 2020 and June 2021 among patients in waiting rooms of 6 public clinics and 2 private emergency departments in economically and socially diverse urban and suburban areas. Respondents were classified as prior nonprescription users if they reported previously taking oral antibiotics without contacting a doctor, dentist, or nurse. Intended use was defined as answering “yes” or “maybe” to the question, “Would you use antibiotics without contacting a doctor, nurse, or dentist?” We calculated the sensitivity, specificity, and positive and negative predictive value (PPV and NPV) of prior nonprescription antibiotic use in the past 12 months for future intended nonprescription use. Bayes PPV and NPV were also calculated, considering the prevalence of nonprescription antibiotic use (24.8%) in our study. **Results:** Of the 564 patients surveyed, the median age was 51 years (SD, 19–92), with 72% of patients identifying as female. Most were from the public healthcare system (72.5%). Most respondents identified as Hispanic or Latino(a) (47%) or African American (33%), and 57% received Medicaid or the county financial assistance program. Prior nonprescription use was reported by 246 (43%) of 564 individuals, with 91 (16%) reporting nonprescription use within the previous 12 months. Intention to use nonprescription antibiotics was reported by 140 participants (25%). The sensitivity and specificity of prior nonprescription use in the past 12 months to predict the intention to use nonprescription antibiotics in the future were 75.9% (95% CI, 65.3–84.6) and 91.4% (95% CI, 87.8–94.2), respectively. After the Bayes’ adjustment, the PPV and NPV of prior use to predict future intention were 74.5% (95% CI, 66.7–80.9) and 92.0% (95% CI, 88.7–94.4) (Table 1). **Conclusions:** These results show that prior nonprescription antibiotic use in the past 12 months predicted the intention to use nonprescription antibiotics in the future (PPV of 75%). As a stewardship effort, we suggest clinicians use a simple question about prior nonprescription antibiotic use in primary-care settings as a screening question for patients at high risk for future nonprescription antibiotic use.

**Figure:**
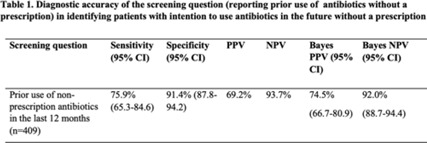

**Financial support:** HSQR-R 5R01HS026901-04

**Disclosure:** None

